# Randomized, Double-Blind Assessment of LFP Versus SUA Guidance in STN-DBS Lead Implantation: A Pilot Study

**DOI:** 10.3389/fnins.2020.00611

**Published:** 2020-06-12

**Authors:** Musa Ozturk, Ilknur Telkes, Joohi Jimenez-Shahed, Ashwin Viswanathan, Arjun Tarakad, Suneel Kumar, Sameer A. Sheth, Nuri F. Ince

**Affiliations:** ^1^Department of Biomedical Engineering, University of Houston, Houston, TX, United States; ^2^Department of Neuroscience and Experimental Therapeutics, Albany Medical College, Albany, NY, United States; ^3^Department of Neurology, Icahn School of Medicine at Mount Sinai, New York, NY, United States; ^4^Department of Neurosurgery, Baylor College of Medicine, Houston, TX, United States; ^5^Department of Neurology, Baylor College of Medicine, Houston, TX, United States

**Keywords:** Parkinson’s disease, subthalamic nucleus, single unit activity, local field potentials, electrophysiological targeting

## Abstract

**Background:** The efficacy of deep brain stimulation (DBS) therapy in Parkinson’s disease (PD) patients is highly dependent on the precise localization of the target structures such as subthalamic nucleus (STN). Most commonly, microelectrode single unit activity (SUA) recordings are performed to refine the target. This process is heavily experience based and can be technically challenging. Local field potentials (LFPs), representing the activity of a population of neurons, can be obtained from the same microelectrodes used for SUA recordings and allow flexible online processing with less computational complexity due to lower sampling rate requirements. Although LFPs have been shown to contain biomarkers capable of predicting patients’ symptoms and differentiating various structures, their use in the localization of the STN in the clinical practice is not prevalent.

**Methods:** Here we present, for the first time, a randomized and double-blinded pilot study with intraoperative online LFP processing in which we compare the clinical benefit from SUA- versus LFP-based implantation. Ten PD patients referred for bilateral STN-DBS were randomly implanted using either SUA or LFP guided targeting in each hemisphere. Although both SUA and LFP were recorded for each STN, the electrophysiologist was blinded to one at a time. Three months postoperatively, the patients were evaluated by a neurologist blinded to the intraoperative recordings to assess the performance of each modality. While SUA-based decisions relied on the visual and auditory inspection of the raw traces, LFP-based decisions were given through an online signal processing and machine learning pipeline.

**Results:** We found a dramatic agreement between LFP- and SUA-based localization (16/20 STNs) providing adequate clinical improvement (51.8% decrease in 3-month contralateral motor assessment scores), with LFP-guided implantation resulting in greater average improvement in the discordant cases (74.9%, *n* = 3 STNs). The selected tracks were characterized by higher activity in beta (11–32 Hz) and high-frequency (200–400 Hz) bands (*p* < 0.01) of LFPs and stronger non-linear coupling between these bands (*p* < 0.05).

**Conclusion:** Our pilot study shows equal or better clinical benefit with LFP-based targeting. Given the robustness of the electrode interface and lower computational cost, more centers can utilize LFP as a strategic feedback modality intraoperatively, in conjunction to the SUA-guided targeting.

## Introduction

Deep brain stimulation (DBS) is an effective treatment option for patients suffering from various neurological disorders such as Parkinson’s disease (PD) (Benabid et al., 2008; Groiss et al., 2009; Schiefer et al., 2011; Hariz, 2012, 2014; Odekerken et al., 2013; Gunduz et al., 2017; Lee et al., 2018). Although the exact mechanism of DBS remains to be explored, it is well-established that stimulation of the subthalamic nucleus (STN) alleviates the cardinal symptoms of PD (Limousin et al., 1998; Krack et al., 2003; Herzog et al., 2004; Benabid et al., 2009). However, stimulation can also result in side effects arising from unintended activation of structures surrounding the STN (Krack et al., 2001; Okun et al., 2003; Deuschl et al., 2006; Guehl et al., 2006; Wojtecki et al., 2007; Benabid et al., 2009; Groiss et al., 2009; Richardson et al., 2009; Zhang et al., 2016). Moreover, a multi-center study has reported that the sub-optimal positioning of DBS electrodes accounts for 46% of cases with inadequate clinical improvement postoperatively (Okun et al., 2005). Thus, the clinical efficacy of DBS therapy depends critically on accurate localization of the STN (Zonenshayn et al., 2000; Sterio et al., 2002; Amirnovin et al., 2006; Gross et al., 2006; Campbell et al., 2019).

Precise placement of the DBS lead can be challenging due to the small size and the anatomical variability in the human STN (Patel et al., 2008; Richardson et al., 2009). While MRI-guided asleep DBS is being performed by some centers (Aziz and Hariz, 2017; Brodsky et al., 2017; Chen et al., 2018; Ho et al., 2018; Wang et al., 2019; Liu et al., 2020), intraoperative electrophysiology remains to be an important technique for localizing the STN, despite the variations in the surgical procedure between medical centers (Zonenshayn et al., 2000; Sterio et al., 2002; Amirnovin et al., 2006; Gross et al., 2006; Abosch et al., 2013; Campbell et al., 2019). A worldwide survey involving 143 DBS centers reported that 83% of them use single unit activity (SUA) recordings for DBS lead implantation (Abosch et al., 2013). Typically, up to five microelectrodes are advanced toward the target structure to obtain a 3-dimensional perspective (Gross et al., 2006; Benabid et al., 2009; Abosch et al., 2013). SUA is used to identify cells with firing characteristics consistent with STN neurons and response characteristics confirming the motor sub-territory of the STN based on a variety of visual and auditory cues (Hutchison et al., 1998; Magnin et al., 2001; Rodriguez-Oroz et al., 2001; Abosch et al., 2002; Benazzouz et al., 2002). This procedure is subjective, heavily experience-based and depends critically on the neurosurgeon’s or electrophysiologist’s ability to recognize the STN (Benazzouz et al., 2002; Benabid et al., 2009; Marceglia et al., 2010; Abosch et al., 2013). Aside from difficulties in interpreting the data and small number of neurons sampled by 1–5 microelectrodes, challenges in interface stability (e.g., necessity of turning lights or other devices off in the operating room) and high bandwidth/sampling frequency requirements may complicate the collection and real-time analysis of SUA (Novak et al., 2011; Rouse et al., 2011; Thompson et al., 2014).

Local field potentials (LFPs), which represent the aggregated synaptic potentials of a population of neurons (Priori et al., 2004; Gross et al., 2006; Buzsáki et al., 2012), can be obtained from the shaft of the same microelectrode used for SUA recordings. Although LFPs have been shown to contain biomarkers capable of predicting Parkinsonian symptoms (Foffani et al., 2003; Ray et al., 2008; Lopez-Azcarate et al., 2010; Özkurt et al., 2011; Little and Brown, 2012; Oswal et al., 2013; Priori et al., 2013; Brittain and Brown, 2014; Ozturk et al., 2019) and differentiating basal ganglia structures (Chen et al., 2006; Telkes et al., 2016; Kolb et al., 2017) only a handful of centers around the world rely on LFPs for the localization of the STN (Abosch et al., 2013).

Here, we present, for the first time, a randomized, double-blinded study comparing the targeting performance of SUA- vs LFP-based implantation. While SUA was interpreted by visual and auditory inspection of the raw traces as done in clinical practice, we employed real-time intraoperative processing of LFPs to facilitate the selection of the implantation track.

## Patients and Methods

### Patients

Ten patients (four females, six males) with PD undergoing bilateral STN-DBS implantation at Baylor St. Luke’s Medical Center were included in the study. Their ages ranged between 40 to 64 (mean ± standard deviation = 55 ± 8.8) with disease duration ranging from 4 to 16 years (mean ± standard deviation = 9 ± 3.9). Nine patients were implanted with Medtronic lead model 3389, and one was with model 3387 (Medtronic, Ireland). The study protocol was approved by the Institutional Review Boards of Baylor College of Medicine and University of Houston. All patients provided written informed consent.

### Study Design

**FIGURE 1:**
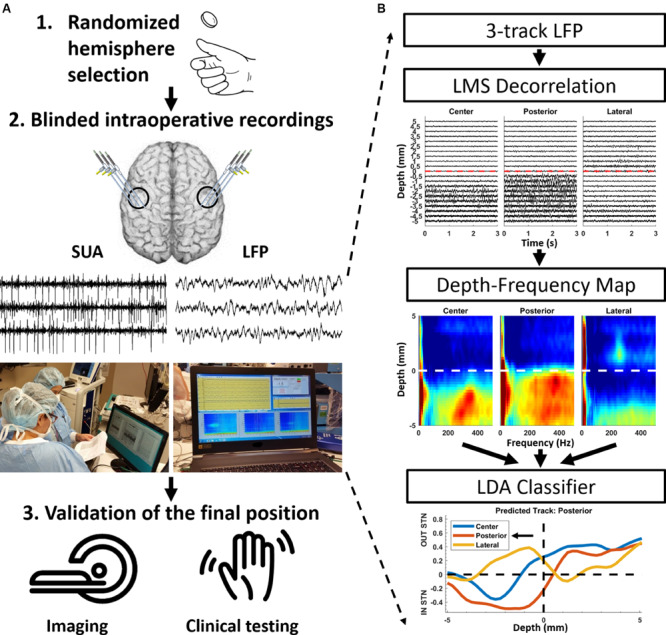
Randomized double-blinded paradigm and the processing pipeline for LFPs. **(A)** The decision modality for each hemisphere was determined in the beginning of the operation randomly. Intraoperative recordings included both SUA and LFP activity. However, only the decision modality, either SUA or LFP, was made available to the electrophysiologist. After the implantation site was selected, the final position was confirmed with intraoperative imaging and clinical testing for motor improvement and side effects, which was performed by a neurologist blinded to the electrophysiological recordings. **(B)** Signal processing and machine learning pipeline for LFP-based decision making. The raw traces are de-correlated using least mean square (LMS) algorithm with the steepest descent update. Then, the LFP traces were analyzed in the spectral domain using modified Welch periodogram with a 1 s Hamming window and 50% overlap. Individual spectra across depths were combined to generate a 2D depth-frequency map (DFM) representing the depth-varying power spectrum of the LFPs. The track selection was performed automatically using a linear discriminant analysis (LDA) classifier developed by Telkes et al. (2016), using the power in beta and HFO bands as input features.

This study investigates the functional utility of LFP versus SUA in targeting the STN with an online processing pipeline (Figure 1A) and compares both modalities in terms of clinical outcomes postoperatively. The implantation modality for each hemisphere (SUA vs. LFP) was randomly identified prior to the surgery. If one hemisphere was implanted using LFP, the other one was implanted using SUA. Three track MER was performed with only the guiding waveform provided to the electrophysiologist for decision making, while the other signal was recorded in the background (blinded recordings) for off-line comparison. After DBS lead placement in the selected track, an intraoperative computed tomography (CT) fused with preoperative magnetic resonance imaging (MRI) was used to verify lead location. Finally, a neurologist blinded to the recordings tested the patients for clinical benefit and side effects intraoperatively and 3-months postoperatively (blinded testing). To prevent possible interference induced by inter-rater variability on the paired statistics performed in this study, the rating neurologists (authors JS and AT, both MDS-UPDRS certified) performed the clinical assessment for each patient consistently (the same rater performed both OFF and ON assessments of a patient, for both the left and right hemispheres). The systematic testing done at 3-months postoperatively was used to assess the clinical improvement by stimulation (medication OFF/DBS ON). The clinical scores were computed as the sum of Movement Disorders Society Unified Parkinson’s Disease Rating Scale (MDS-UPDRS) Part-III items 3.3–3.8, 3.15–3.17 of the side contralateral to the implant.

### Intraoperative Recordings

Patients were requested to stop medication at least 12 h prior to surgery and all recordings were obtained in the awake state using local anesthesia. On the morning of the surgery, all patients obtained a head CT after application of the stereotactic head frame. The stereotactic coordinates and trajectories to the STN were identified by fusing preoperative MRI and CT scans on a neuro-navigational platform (StealthStation, Medtronic, Ireland). In each hemisphere, awake recordings were performed using a set of three parallel microelectrodes separated by 2 mm (center-to-center) using the 5-cannula BenGun with “+” configuration. The preoperative planning using direct targeting methods determined the “center” track. Among “anterior, posterior, lateral and medial” tracks, two other tracks were selected by the neurosurgeon on a patient specific basis by taking into account the subject’s anatomy. The microelectrodes (NeuroProbe, AlphaOmega, Israel) were initially placed at least 15 mm above the stereotactic target and advanced deeper with 0.5–1 mm steps using NeuroOmega drive (AlphaOmega, Israel), in order to refine the radiographic target. At each depth, by using the cannula as reference, at least 20 s of SUA from the high-impedance tungsten tip (0.6–0.8 MΩ) and LFP from the low impedance stainless-steel ring (<10 KΩ, 3 mm above the tip) on the shaft were obtained simultaneously. The entire data was recorded with Grapevine Neural Amplifier (Ripple Neuro, UT) at 30 KHz and 16-bit A/D resolution, and LFPs were down-sampled to 2 KHz before further processing.

### Signal Processing

**FIGURE 2:**
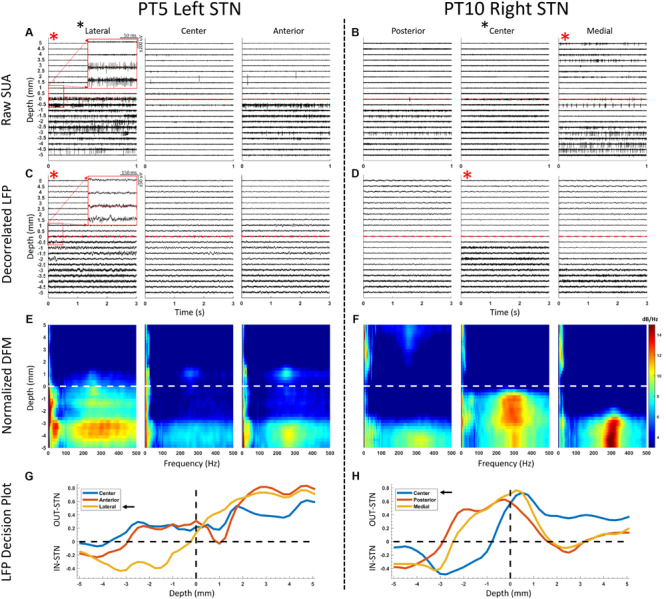
Representative SUA and LFP raw traces and depth-frequency maps (DFM) from two STNs where the suggested track by SUA and LFP overlapped (left panel) and did not overlap (right panel). **(A,B)** SUA raw traces presented from 5 mm above down to 5 mm below the dorsal border of the STN, with 0.5 mm steps. The dorsal border is marked by an increased spiking and background activity. Red asterisks denote suggested track by the corresponding modality and black asterisk next to the track name denotes the implanted track. **(C,D)** The decorrelated LFP traces are provided in a similar fashion to SUA traces. Although it can be observed that the oscillatory activity increases after crossing the dorsal border, the nature of the change can be visualized better in the spectral domain where **(E,F)** DFMs of the corresponding LFPs are presented. The entry to STN is characterized with increased activity in both beta and HFO ranges. **(G,H)** Decision plots of the classifier voting whether each track is in- or out-STN at each traversed depth. The track with the longest in-STN vote is selected as the implanted track, as indicated with the black arrows. The SUA activity was stronger in the lateral track **(A)** and the corresponding LFPs agreed **(G)** in the left panel. On the right panel, SUA **(B)**, which was the decision modality, suggested the medial track. However, after intraoperative imaging validation the lead was placed in the center track, which agreed with the LFP-based decision **(H)**.

The signals were recorded and visualized in real-time with an in-house built Simulink model and processed with custom MATLAB scripts using version R2014a (Mathworks, Natick, MA, United States) and gHiSys high-speed online processing library (gTec, Austria). The entire online processing was performed on a 17” laptop with quad-core (2.4 GHz) processor and 12 GB memory. The SUA data were high-pass filtered at 300 Hz with a second order infinite-impulse response filter and presented to the electrophysiologist in visual and auditory format, similar to the commercially available devices. After the mapping was completed, the SUA traces were plotted depth by depth from all three tracks for reviewing and final decision. The entry to and exit from the STN was determined by an experienced neurophysiologist by listening to and visually observing the firing patterns of neurons. The entry to the STN was identified with a prominent increase in the background activity and discharge rates (Figure 2A), as reported previously (Hutchison et al., 1998; Novak et al., 2007). Among three, the track with the longest span of cell firing and background activity was selected for the chronic DBS electrode implantation (Abosch et al., 2002; Benazzouz et al., 2002; Gross et al., 2006). In those hemispheres where the implantation was performed based on LFPs, the same procedures were used to process SUA data offline, following the implantation of the DBS electrode.

The LFPs were processed intraoperatively with the real-time implementation of the signal processing pipeline (Figure 1B) provided by Telkes et al. (2016). Specifically, LFP raw traces were visualized initially and it was noted that tracks were difficult to distinguish, due to common activity coming from the reference contact (cannula) masking spatially localized patterns. In order to eliminate the common activity without affecting the localized neural activity, the LFP from tracks were de-correlated using a least mean square (LMS) algorithm with the steepest descent update. Explicitly, each track was predicted by using a linear weighted combination of other two channels and the residual was used for the further processing. With this adaptive approach, the common activity was eliminated across tracks and only spatially specific information was preserved (Telkes et al., 2016). LFP traces were then analyzed in the spectral domain using a modified Welch periodogram. A fast Fourier transform was computed at each depth with a 1 s Hamming window and 50% overlap and presented to the electrophysiologist in near real-time in the form of online spectrograms (Supplementary Video S1). After the mapping was completed, a median spectrum was calculated from the spectra to eliminate localized artifacts at each depth. Then, spectra across depths were combined to generate a 2D depth-frequency map (DFM) representing the depth-varying power spectrum of the LFPs of each track (Telkes et al., 2016, 2018). The maps were then normalized with the average baseline of three tracks and transformed into log scale (Figure 1B). The tracks were not normalized by their own baseline but by the mean of all three tracks in order to compare the signal power between them. The baseline used for normalization was selected as the highest depths which are assumed to be in the white matter. The STN was identified by distinct LFP activity in beta (11–32 Hz) and HFO (200–400 Hz, high-frequency oscillations) ranges. The track containing the largest beta and HFO bandpower for the longest span was selected as the implantation site for the DBS electrode (Zaidel et al., 2010; Wang et al., 2014; Telkes et al., 2016; van Wijk et al., 2017). This selection was performed automatically using a linear discriminant analysis (LDA) classifier developed by Telkes et al. (2016). Specifically, after obtaining the normalized depth-frequency maps, the beta and HFO sub-band powers were extracted for each track and depth from these maps. Then, the sub-band power features were normalized between zero and one with a Min-Max normalization method for the minimization of inter-subject variability in LFP power, and a binary LDA classifier was applied for classification. This classifier was trained by contrasting the LFP sub-band features coming from selected and non-selected tracks using the data from the 24 PD patients analyzed in Telkes et al. (2016). During online classification, the neural data in each track and at each depth were fed to the classifier. Therefore, each electrode trajectory received a vote at each depth from the classifier. The decision distance of the LDA classifier was plotted to give visual feedback regarding the votes and related confidence of the classifier (Figure 1B). The track that received the longest span of decision distances voting for in-STN was selected for the final DBS electrode implantation (Figure 1B). Once again, in those hemispheres where the implantation was performed based on SUA, the same signal processing pipeline was executed offline to process LFPs, following the implantation of the DBS electrode.

Additional offline analysis was performed postoperatively to investigate the cross-frequency coupling (CFC) between beta and HFO bands. The comodulograms representing CFC were computed using the phase-locking principle (Penny et al., 2008) with amplitude frequency axis from 150 to 450 Hz with 10 Hz steps and 50 Hz filter bandwidth, and phase frequency axis from 6 to 40 Hz with 1 Hz steps and 3 Hz filter bandwidth.

### Statistics

Normality of all distributions was tested using Anderson-Darling test and it was found that most of them are non-normal (*p* < 0.05). Statistical tests were performed in a paired fashion using non-parametric Wilcoxon signed rank test to compare the clinical scores in the OFF- and ON- DBS states, the beta and HFO bandpowers and the coupling strength between them. The sample size and significance levels are provided throughout the text, when referred.

## Results

A total of 60 microelectrode tracks from 20 STNs were included in this study. Figure 2 illustrates offline comparison of representative LFP and SUA data from two STNs, where both modalities suggested the same track in one (Pt5, left hemisphere) and different tracks in another (Pt10, right hemisphere). The SUA-predicted tracks were determined by the longest span of background and spiking activity (Figures 2A,B) whereas the longest span of in-STN votes of the classifier were considered in LFP-based selection (Figures 2C–F). The decision distance (y-axis of Figures 2G,H) represented the confidence of the classifier which used the power in beta and HFO bands of LFP as input features. Note that, although the randomized decision modality was SUA for the right hemisphere of patient 10 (Figure 2, right panel) suggesting implantation in the medial track, intraoperative CT favored the center track as the target, which was used as the final implantation location. The offline analysis of LFPs agreed with the radiographic decision as well.

**FIGURE 3:**
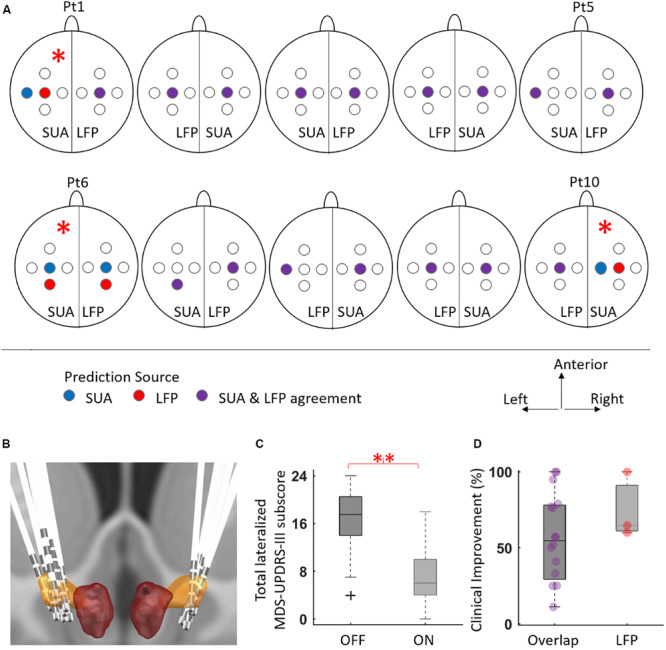
The distribution of selected tracks by SUA and LFP, and the corresponding motor improvement. **(A)** The BenGun representation of suggested and implanted tracks for individual STNs. For each hemisphere, the randomized implantation modality is written at the bottom. There were three cases where the decision modality was SUA, but the lead had to be repositioned (denoted by asterisk): left hemisphere of patient 1 moved to posterior track due to intraoperative stimulation side effects, without use of electrophysiology (excluded from further analysis); left hemisphere of patient 6 and right hemisphere of patient 10 repositioned due to discrepancy with the intraoperative imaging. In the latter two cases, the LFP suggested track agreed with the final decision. **(B)** The illustration of implanted DBS leads generated by merging preoperative MRI and intraoperative CT using Lead DBS toolbox (Horn and Kühn, 2015). In all hemispheres, at least one contact was observed to be in STN. **(C)** The distribution of total contralateral motor UPDRS scores in the DBS OFF and DBS ON states for 19 hemispheres. There was a significant clinical motor improvement (51.8%) after DBS treatment (*p* < 0.01, *n* = 19). **(D)** The tracks suggested by SUA and LFP overlapped for 16 STNs, with average improvement of 55.5%. When there was a disagreement and the implant location agreed with LFP (*n* = 3), the average improvement was 74.9%. Individual data points are presented with circles to emphasize the unequal sample size between groups.

Distribution of decisions for all hemispheres given by each modality as well as their randomization is provided in Figure 3A. In 16/20 hemispheres, the SUA and LFP recordings were concordant in their prediction of implantation track. In those four discordant hemispheres, the LFP was the decision modality in only one of them and the final implantation validated by intraoperative CT and clinical testing agreed with LFP-suggested track. In remaining three STNs where SUA was the decision modality, the lead had to be repositioned based on intraoperative CT validation and/or stimulation testing. For two STNs (Pt6, left; Pt10 right), the track suggested by the SUA, did not agree with the track residing within the target confirmed radiographically (according to intraoperative CT scans merged with preoperative MRI). Therefore, the DBS electrode was placed into the most likely track suggested by the radiography. Interestingly, for these two STNs, the track suggested by the imaging agreed with LFPs. In one STN (Pt1, left), the lead was moved to posterior track due to stimulation side-effects during intraoperative testing and imaging considerations, without the use of microelectrode recordings. This STN was excluded from further analysis. The repositioned hemispheres are marked with a star on Figure 3A. Overall, the track favored by SUA was implanted in a total of 16 chronic lead placements whereas LFP-favored track was used in 19. In addition to intraoperative radiographic validation, all 20 implantations were visualized postoperatively by merging the preoperative MRI and postoperative CT images using LeadDBS (Horn and Kühn, 2015). It was observed that at least one contact of the DBS lead was within the STN (Figure 3B).

The mean lateralized MDS-UPDRS part III improvement for 19 STNs was 51.8% at 3-month postoperative programming (mean ± standard deviation OFF score = 16.3 ± 5.4, ON score = 6.5 ± 4.6, Figure 3C). When the track decisions were compared across modalities in terms of outcome measures, the 16 STNs where both modalities agreed had average clinical improvement of 55.5%. Of the tracks with LFP-SUA mismatch, the mean improvement in three LFP-concordant implantations was 74.9% (Figure 3D).

**FIGURE 4:**
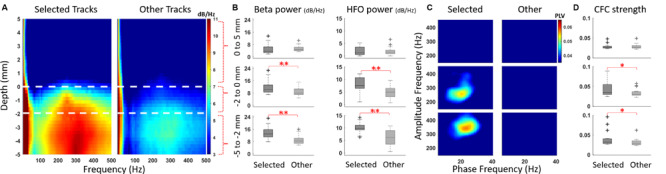
LFP patterns of selected and non-selected (other) tracks. **(A)** DFM averages of LFPs recorded from selected and other tracks 5 mm above and below the dorsal border of 19 STNs. One hemisphere which was repositioned due to side effects without recordings was excluded from the electrophysiological averages. The white dashed lines represent the dorsal border and, the dorsal and ventral regions of STN. Upon entry to the STN, there was a marked increase in the beta and HFO activity. The dorsal half was dominated with slow HFOs (200–280 Hz) whereas the ventral half had HFOs in the 280–400 Hz range. **(B)** The beta and HFO bandpowers are contrasted between selected and other tracks in three spatial regions: above (5–0 mm), dorsal (0 to -2 mm) and ventral (-2 to -5 mm) STN. There was significant difference in both beta and HFO bandpower in dorsal and ventral regions (*p* < 0.01, *n* = 19). **(C)** The CFC patterns contrasted from the same depth regions revealed beta-slow HFO coupling in the dorsal territory, whereas there was beta-fast HFO coupling in the ventral territory of the selected track. **(D)** The mean beta-HFO coupling strength was significantly higher in both dorsal and ventral territories (*p* < 0.05, *n* = 19).

The average DFMs and CFC comodulograms of selected vs other tracks from 19 STNs are presented in Figure 4. The left hemisphere of patient 1 was excluded since the electrode was repositioned due to side effects observed during intraoperative stimulation testing without neural recordings. The STN was characterized by exaggerated activity in the beta and HFO ranges in the selected track while the average map of the non-selected tracks contained weaker beta and HFO activity, as presented in Figure 4A. The power of these two bands were significantly higher in the selected track, both in dorsal and ventral regions (Figure 4B, *p* < 0.01, *n* = 19). Although three patients had localized HFO activity above the STN border (see representative DFMs in Figures 2E,F), there was no significant difference in HFO bandpower between selected and other tracks at this depth range. The dorsal half of STN demonstrated CFC between the phase of beta and the amplitude of slow HFO (200–280 Hz) oscillations whereas the ventral half was coupled with fast HFO (280–400 Hz) band as illustrated in Figure 4C. Amongst all selected tracks, the beta-HFO coupling strength was significantly higher in both dorsal and ventral territories, when compared to other tracks (Figure 4D, *p* < 0.05, *n* = 19).

## Discussion

In this blinded study, we compared the functional utility of LFPs for the implantation of DBS electrode against the widely used method, SUA (Benabid et al., 2009; Przybyszewski et al., 2016; Valsky et al., 2017). We observed an overall agreement in track prediction between both modalities (16/20 hemispheres) with adequate clinical benefit (55.5%) from chronic DBS, comparable to previous reports (Limousin et al., 1998; Krack et al., 2003; Walter and Vitek, 2004). In the three discordant cases, our findings suggest that the mean improvement in motor symptoms with LFP guided implantation may be greater.

The large overlap between optimal tracks predicted by both SUA and LFP is not a surprise as firing activity and field potentials have shown to be linked (Kühn et al., 2005; Buzsáki et al., 2012; Telkes et al., 2016; Meidahl et al., 2019), and supports the use of LFP-guided lead placement. A possible explanation for the mismatched hemispheres could be the stability issues in the electrode tip - tissue interface (Amirnovin et al., 2006; Hill et al., 2011; Harris et al., 2016). In one STN presented in Figure 2B, although the background activity in center track SUA increases after the border (0 mm), a potential tip failure (i.e., bending or damage to the fine tip of microelectrode that could reduce the high impedance, which is essential to capture SUA) could have prevented the isolation of individual neurons. Since the LFP traces (Figure 2D) and DFM (Figure 2F) of the same STN show strong activity correlated with intraoperative CT, a technical or hardware issue specific to the tip of the microelectrode is a distinct possibility. Even without any damage, the SUA tip may not necessarily isolate single neurons at every site (Benazzouz et al., 2002; Weinberger et al., 2006; Sharott et al., 2014) by being too far to the cells or by damaging them (Buzsáki, 2004; Harris et al., 2016). In such instances, the electrophysiologist faces the uncertainty of missing the target or missing the neurons. By contrast, the stainless-steel ring on the shaft where LFPs are recorded has more structural integrity, larger surface and smaller impedance (Lenz et al., 1988; Gross et al., 2006), and captures the oscillatory activity from a population of neurons (Priori et al., 2004; Gross et al., 2006; Buzsáki et al., 2012), thereby limiting the chances of missing the electrophysiological activity (Buzsáki et al., 2012; Priori et al., 2013; Thompson et al., 2014). Supporting the favorability of LFP recordings, we found that among three cases where SUA was the deciding modality but the implantation track had to be modified, two of the final locations agreed with the LFP-based track selection (Figure 3A).

Intraoperative electrophysiological recordings for the accurate localization of STN have been a vital step for DBS electrode implantation (Zonenshayn et al., 2000; Sterio et al., 2002; Amirnovin et al., 2006; Gross et al., 2006; Abosch et al., 2013; Campbell et al., 2019). SUA has been the most commonly used electrophysiological signal for targeting (Gross et al., 2006; Abosch et al., 2013; Campbell et al., 2019), which strongly relies on subjective interpretation of single unit firings (Benazzouz et al., 2002; Benabid et al., 2009; Marceglia et al., 2010; Abosch et al., 2013). Recently, there have been reports to ameliorate this disadvantage by identifying and clustering firing types (Kaku et al., 2019, 2020) or by detecting entry and exit of the STN automatically (Wong et al., 2009; Zaidel et al., 2009; Pinzon-Morales et al., 2011; Valsky et al., 2017; Thompson et al., 2018). However, the volatile interface stability and increased computational power requirement arising from higher sampling rates might still favor LFPs (Rouse et al., 2011; Buzsáki et al., 2012; Priori et al., 2013; Thompson et al., 2014). Growing literature supports the utility of LFPs in intraoperative mapping (Chen et al., 2006; Przybyszewski et al., 2016; Telkes et al., 2016; Kolb et al., 2017; Lu et al., 2019). Our results also support the use of LFPs intraoperatively for DBS lead implantation. The processing pipeline and real-time visualization tool (Supplementary Video S1) presented here can facilitate this process.

The exploration of disease biomarkers for the development of novel technologies such as closed loop DBS have been of great interest lately (Little and Brown, 2012; Priori et al., 2013; Meidahl et al., 2017; Hell et al., 2019). In this regard, LFPs can provide variety of non-binary patterns including power of distinct oscillatory bands and their nonlinear interactions. There is an abundance of studies reporting the response of LFP-derived biomarkers to medication (Foffani et al., 2003; Priori et al., 2004; Marceglia et al., 2006; Kane et al., 2009; Lopez-Azcarate et al., 2010; Özkurt et al., 2011; Ozturk et al., 2019) and DBS (Kühn et al., 2008; Eusebio et al., 2011; McConnell et al., 2012) therapies, as well the correlation between these biomarkers and cardinal symptoms of PD (Kühn et al., 2006; Weinberger et al., 2006; Ray et al., 2008; Lopez-Azcarate et al., 2010; Oswal et al., 2013; Brittain and Brown, 2014; Ozturk et al., 2019). We and others have previously shown that these patterns can provide utility in contact selection (Ince et al., 2010; Connolly et al., 2015) or targeting the optimal location for DBS implantation (Chen et al., 2006; Thompson et al., 2014; Telkes et al., 2016; Kolb et al., 2017; Lu et al., 2019). Specifically, oscillations in the beta and HFO range and their cross-frequency interactions have been used to pinpoint the “sweet spot” for DBS (Wang et al., 2014; Connolly et al., 2015; Telkes et al., 2016; Horn et al., 2017; van Wijk et al., 2017; Hell et al., 2019). When comparing the selected track with others, we have observed that the bandpowers of beta and HFO oscillations were significantly higher in both dorsal and ventral parts of the STN. Postoperative analyses revealed that the coupling pattern between phase of beta and amplitude of HFO differed in dorsal and ventral territories, similar to previous reports distinguishing both regions with electrophysiology (Rodriguez-Oroz et al., 2001; Theodosopoulos et al., 2003; Zaidel et al., 2009; Telkes et al., 2018). This difference is expected as the dorsolateral STN has been associated with motor functions and exhibited distinct oscillatory/bursting single unit firings whereas ventromedial STN is associated with limbic functions and tonic firings (Abosch et al., 2002; Gross et al., 2006; Zaidel et al., 2010; Thompson et al., 2018; Campbell et al., 2019; Kaku et al., 2020). Interestingly, we also noted HFOs above STN in three patients (see Figure 2E). This activity could be originating from other structures such as thalamus or zona incerta (ZI) (Thompson et al., 2014; Yang et al., 2014; Lu et al., 2019; Meidahl et al., 2019). Previous work has shown that dorsolateral STN and ZI stimulation provides the greatest improvement in PD motor symptoms (Gourisankar et al., 2018), which correlates with our observation. However, lack of activity in the bulk of our recordings begs for further investigation regarding the out-of-STN oscillatory activity in more patients.

## Conclusion

In this report, we assessed the functional utility of LFP-based lead implantation against the gold standard SUA method using intraoperative online signal processing and compared these modalities in terms of clinical outcomes. Our results suggest that the LFP oscillations can be a more stable, less processing-intensive method that can be integrated in the intraoperative workflow together with SUA-based mapping, without affecting the surgical procedure. In addition to the functional role of LFPs in intraoperative target mapping, the fact that LFPs can also be recorded from the chronic DBS lead after the surgery is another potential advantage that might guide therapeutic programming to a higher efficacy and efficiency. Here, we provided results of a pilot study with ten patients. Future clinical trials with more subjects will be needed to establish if LFPs can become the standard of care for intraoperative mapping.

## Data Availability Statement

The data that support the findings of this study are available on a reasonable request from the corresponding author. The raw data are not publicly available as the data might contain potentially identifying or sensitive information that could compromise the privacy of the research participants.

## Ethics Statement

The studies involving human participants were reviewed and approved by University of Houston and Baylor College of Medicine. The patients/participants provided their written informed consent to participate in this study. Written informed consent was obtained from the individual(s) for the publication of any potentially identifiable images or data included in this article.

## Author Contributions

AV, NI, and JJ-S conceived and designed the experiments. MO, IT, and AV performed the experiments. JJ-S and AT performed clinical testing. MO and NI analyzed the data. MO, IT, and NI wrote the manuscript. MO, IT, JJ-S, AT, AV, SS, SK, and NI reviewed and revised the manuscript and approved the final manuscript as submitted.

## Conflict of Interest

The authors declare that this study received funding from Medtronic. The funder was not involved in the study design, collection, analysis, interpretation of the data, the preparation of this manuscript or the decision to submit for publication.

## Supplementary Material

VIDEO S1 The soft real-time processing of the LFP activity from 3-track microelectrode recordings (playback accelerated by x50). The traces of ECG, upper limb EMG and LFP signals are provided at the top. The time-evolving frequency spectrum in the low (0–60 Hz) and high (60–500 Hz) frequency ranges of each LFP track is provided at the bottom. As the time passes, the electrodes are inserted deeper towards the target.
